# MiR-187 suppresses non-small-cell lung cancer cell proliferation by targeting FGF9

**DOI:** 10.1080/21655979.2019.1706287

**Published:** 2019-12-28

**Authors:** Zhihua Liang, Jianhui Xu, Zhancheng Ma, Guihua Li, Wanhong Zhu

**Affiliations:** a Department of Respiratory, HeXian Memorial Hospital Affiliated with Southern Medical University, Guang zhou, China; b GuangZhou Chest Hospital, Guang zhou, China

**Keywords:** NSCLC, miR-187, proliferation, FGF9, cyclin D1

## Abstract

Non-small-cell lung cancer (NSCLC) is the main pathological type of lung cancer and has a low overall five-year survival rate. miR-187 has been reported to play major roles in various tumor types. In this study, we explored the impact of miR-187 on NSCLC. qRT-PCR results demonstrated that miR-187 expression is lower in NSCLC and cancer cells than normal tissues and normal lung cells. miR-187 expression levels are associated with tumor size, TNM stage and overall survival rate. MTS and colony formation assays showed that high miR-187 expression inhibits NSCLC cell proliferation and colony formation ability, and flow cytometry showed that miR-187 overexpression induces cell cycle arrest at the G0/G1 phase. A luciferase reporter assay showed that FGF9 is a target of miR-187. miR-187 overexpression reduces the expression of FGF9, cyclin D1 CDK4 and CDK6. Therefore, miR-187 may present a new NSCLC treatment target by regulates cyclins-related protein expression.

## Graphical Abstract

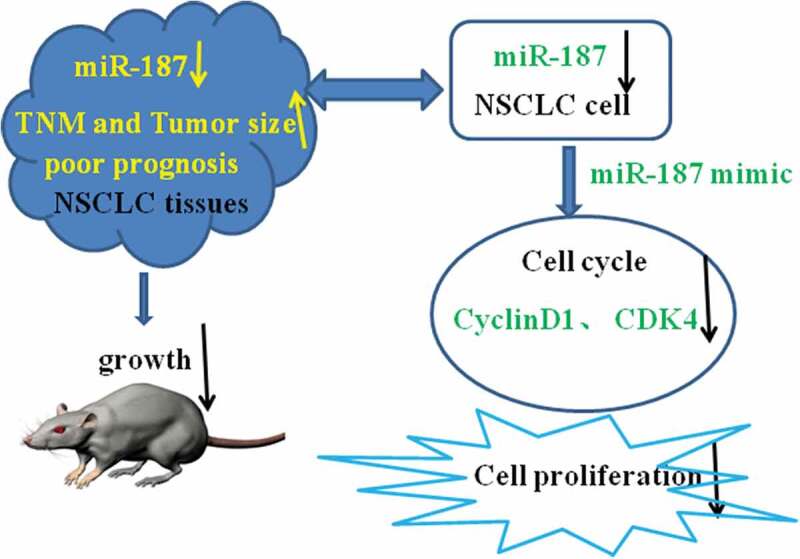

## Introduction

Lung cancer is the most common malignancy worldwide and remains the leading cause of cancer-related mortality, accounting for approximately 25% of all cancer deaths [1,2]. Lung cancer could be divided into two subtypes: small-cell lung cancer and non-small-cell lung cancer (NSCLC). NSCLC is the main pathological type, accounting for 80–85% of lung cancer cases, with a low overall five-year survival rate of 10–15% [3,4]. Despite the development of medical technology, the prognosis for NSCLC still remains poor [5], and the mechanisms underlying the development and progression of NSCLC are not yet clear. The clarification of the mechanisms at the molecular level and identification of effective novel therapeutic targets may contribute to improving the prognosis of NSCLC patients.

MicroRNAs (miRNAs) are a endogenous and conserved cohort of single-stranded non-coding small RNAs of 19–25 oligonucleotides in length [6,7]. miRNAs bind to the 3′UTR of the mRNA of their target genes to repress translation or decrease the mRNA stability at the post-transcriptional level [8,9]. More than 2500 types of miRNAs have been shown to affect the expression of approximately 30% of the human protein-encoding genes [10]. mRNAs have been found to be aberrantly expressed in various cancers, and they can regulate numerous crucial biological processes including cell proliferation, apoptosis, differentiation, and metastasis [11–13]. Accumulating evidence has confirmed that mRNAs play an important role in the development and progression of NSCLC, and they have attracted the attention of oncologists as their potential uses for the cancer diagnosis, treatment, and prognosis [14,15].

miR-187 is a recently identified new cancer-associated miRNA [16]. However, the expression level of miR-187 is different in various cancer tissues. Chen *et al.* [17] reported that miR-187 expression was significantly downregulated in gastric cancer, and low expression of miR-187 correlated with cell differentiation, TNM staging and poor prognosis in patients. However, miR-187 expression was found to be significantly increased in the plasma of oral squamous cell carcinoma (OSCC) patients; miR-187 increases OSCC cell oncogenicity and the xenograft metastasis in mice [18]. These data indicate that miR-187 has important functions in cancer development.

According to recent reports, the function of miR-187 in NSCLC is also different [19,20] and may be related to its target genes; however, the precise molecular mechanism by which miR-187 influences NSCLC progression remains largely unknown. Therefore, the purpose of the present study was to explore the effects of changing the miR-187 expression on the cell proliferation of NSCLC cells and to investigate the mechanisms by which novel target genes of miR-187 are regulated. The evidence showed that miR-187 can as a novel therapeutic target for NSCLC.

## Materials and methods

### Tissue samples

Sixty tissue samples from patients with NSCLC and their paired adjacent normal tissues validated by pathologists were obtained from HeXian Memorial Hospital of Guangzhou City (Guangzhou, China) from 2013 to 2017. All patients did not receive chemotherapy, radiotherapy or any other therapy prior to surgery. The patients provided written informed consent and were followed up in detail. Patients with other kinds of cancer or certain systemic diseases (e.g., systemic lupus erythematosus, rheumatoid arthritis or diabetes) were not included. After surgery, all tissues were frozen and stored at liquid nitrogen immediately before being used for RNA extraction and other tests. The processing of all specimens was approved by the Ethics Committees of HeXian Memorial Hospital.

### Cell culture and transfection

The NSCLC lines (A549, H1975, NCI-H460 and SPC-A-1) and human normal lung epithelial cells (16HBE) were purchased from American Type Culture Collection (ATCC, MD, USA). A549 cells were cultured in DMEM F12 medium (Gibco, NY, USA). H1975, NCI-H460 and SPC-A-1 cells were cultured in RPMI-1640 medium (Gibco). All the cells were maintained in medium supplemented with 10% fetal bovine serum (FBS, Gibco) and cultured at 37°C in an atmosphere with 5% CO_2_. miR-187 mimic and negative control (NC) constructs were purchased from GenePharma (Shanghai, China). To assess the effect of miR-187 on cell proliferation, the miR-187 mimic was transfected into A549 and SPC-A-1 cells using Lipofectamine 2000 (Invitrogen, Thermo Fisher Scientific, USA) according to the manufacturer’s protocol.

## Reverse transcription quantitative PCR (qRT-PCR)

In brief, total RNA was extracted from tissues and cell lines using Trizol solution (Invitrogen, Thermo Fisher Scientific, USA) according to the manufacturer’s instructions and reverse transcribed into cDNA using a PrimeScript™ II First-Strand cDNA Synthesis kit (Takara, Japan). qRT-PCR was conducted by using the SYBR Premix Ex Taq (Takara, Japan) for miRNA detection. The relative miRNA expression was calculated according to the 2^−∆∆Ct^ method, with glyceraldehyde 3-phosphate dehydrogenase (GAPDH) used for normalization. The primer sequences were as follows: miR-187 forward, 5′- TCGTGGGTCGTGTCTTGTGTTGC-3′ and reverse, 5′-GCAGGGTCCGAGGTATTC-3′; FGF9 forward, 5′- ATGGCTCCCTTAGGTGAAGTT-3′ and reverse, 5′-CACTTAACAAAAC-3′; GAPDH forward, 5′- GGAGCGAGATCCCTCCAAAAT −3′ and reverse, 5′- AGCGAGCATCCCCCAAAGTT-3′.

## MTS proliferation assay

Cell proliferation was determined by 3-(4,5-dimethylthiazol-2-yl)-5-(3- carboxymethoxyphenyl)-2-(4-sulfophenyl)-2H-tetrazolium assay (MTS, Promega, USA), by following the manufacturer’s instructions. A549 and SPC-A-1 cells were plated in 96-well plates at a density of 2 × 10^3^ cells/well. After 24 h of static culture, the cells were transfected with the miR-187 mimic and NC. After culturing for 24, 48, 72 or 96 h, 30 µl of MTS solution was added to each well, and the plate was incubated for 2 h at 37°C. The absorbance at 490 nm was measured for each well using a spectrophotometer (Coulter Z1, Beckman Coulter, Germany).

## Colony formation assays

A549 and SPC-A-1 cells were plated in 6-well plates at a density of 1 × 10^3^ cells/well. After 24 h of static culture, the cells were transfected with the miR-187 mimic and NC. One week later, the miR-187 mimic- and NC-transfected cells were transfected once more, and cell colony formation was assessed after two weeks. The cells were washed in phosphate-buffered saline (PBS, three times), fixed with 100% methanol for 20 min, stained with hematoxylin (Baso, Taiwan, China) for 5 min, washed with ddH_2_O, aired and colonies were counted.

## Cell cycle assay

A549 and SPC-A-1 cells were plated in 6-well plates at a density of 1 × 10^6^ cells/well. After 24 h of static culture, the cells were transfected with the miR-187 mimic and NC. Then, 48 h later, 1 × 10^6^ transfected cells from each group were washed three times with PBS, fixed in 85% pre-cooling ethanol for 24 h and treated with 500 µl of staining solution (staining buffer 465 µl, 50X RNase A 10 µl, 20X propidium iodide 25 µl) for 30 min at 37°C according to Beyotime Cell Cycle (shanghai, China) manufacturer’s instructions. Flow cytometry (FACS CantoTM II Flow Cytometer, BD Biosciences, USA) was used for detection.

## Dual luciferase activity assay

To verify the direct interaction of miR-187 with the target gene FGF9, human mRNA sequence was cloned into the pMIR-reporter construct (Ambion; Thermo Fisher Scientific, USA). Wild-type and mutant FGF9 mRNA fragments were amplified and sub-cloned into a luciferase reporter. A549 and SPC-A-1cells plated in 24-well plates were co-transfected with a miR-187 mimic or internal control oligonucleotides, followed by pRL-TK (Promega, Madison, USA) and a firefly luciferase reporter using the JetPRIME reagent (Polyplus-transfection) according to the manufacturer’s instructions. After transfection for 48 h, the luciferase activity was detected using a Dual Luciferase reporter Assay System (Promega).

## Western blot analysis

Cells transfected for 48 h were collected and lysed in ice-cold RIPA buffer containing proteinase inhibitor (solarbio, Beijing, China). The lysates were then centrifuged at 14,000 rpm for 30 min at 4°C. The supernatants were collected to quantify the protein concentration by BCA Reagent kit (Thermo Fisher, USA). Fifty micrograms of protein in the lysates of each sample were separated by 10% sodium dodecyl sulfate-polyacrylamide gel electrophoresis and transferred to polyvinylidene difluoride membranes (PVDF: Millipore, USA). After the nonspecific binding was blocked with 8% nonfat milk for 1 h, the membranes were incubated overnight at 4°C with a primary antibody cyclin D1 (Abcam16663, UK), CDK4 (Abcam199728, UK) and CDK6 (Abcam151247, UK). After being washed three times with TBST, the membranes were incubated with a secondary antibody at a 1:8000 dilution (Proteintech, Chicago, USA) and the target proteins were detected using ECL reagents (GE healthcare, USA).

## Nude mice xenograft assay

Four-week-old female BALB/c nude mice were housed in pathogen-free conditions. Mice were randomly divided into two groups with five mice per group, NC group and miR-187 mimic group; each group tumor xenografts were generated by subcutaneously inoculating 1 × 10^7^ NC A549 cells and miR-187 mimic A549 cells. The cells were suspended in 100 μl PBS and injected into the right flank of the nude mice. The tumor volumes of tumor xenografts were measured every four days by a vernier caliper and calculated as follows: Volume (cm^3^) = a(length)b (width)^2^/2. The mice were sacrificed four weeks after tumor cell inoculation, and the tumor xenografts were excised and weighed. All procedures were supported by the Animal Care and Research Committee of Southern Medical University.

## Statistical analysis

Experimental data were expressed as the mean ± SD (standard deviation) and were analyzed using SPSS 17.0 software. The data were handled using GraphPad Prism 5.0. Student’s *t*-test or one-way ANOVA was used to calculate the significant difference. Pearson’s χ^2^ test was performed to analyze the relationship between miR-187 expression and the clinicopathological characteristics of NSCLC patients. Pearson’s correlation was used to analyze the correlation between the expression of miR-187 and FGF9 in NSCLC tissues. Kaplan–Meier analysis and the log-rank test were performed to analyze overall survival rate. *P* < 0.05 indicated statistical significance.

## Results

### Mir-187 expression downregulation in NSCLC

**Figure 1.**
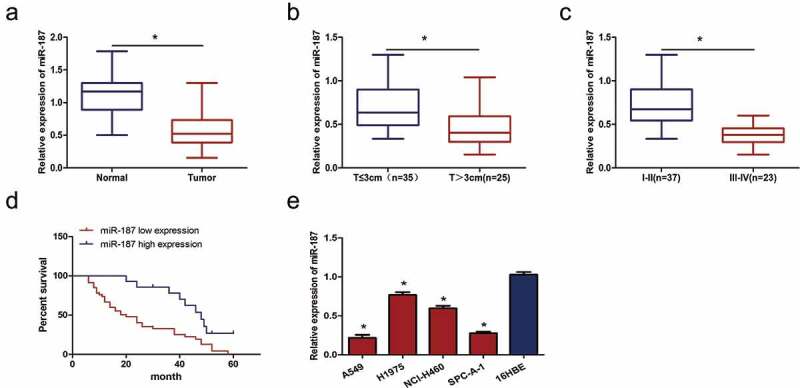
**miR-187 expression is downregulated in NSCLC tissues and cells**. (a) qRT-PCR analysis showed that miR-187 expression is lower in NSCLC tissues than paired normal tissues. (b) miR-187 expression is reduced in NSCLC tissues from large tumors. (c) miR-187 expression is reduced in advanced stages (III-IV) NSCLC tissues. (d) Reduced miR-187 expression is predictive of reduced overall survival rate. (e) miR-187 expression is downregulated in four NSCLC cell lines compared to 16HBE human normal lung epithelial cell; *p < 0.05.

To explore the function of miR-187 in NSCLC, the expression levels of miR-187 in 60 NSCLC patient samples and their paired normal tissues were first examined by using qRT-PCR. miR-187 expression was significantly downregulated in NSCLC cancer tissues compared to normal tissues (*t* = 10.37, *P* < 0.001) (Figure 1(a)). The association between miR-187 expression and the clinicopathological characteristics of NSCLC was explored further. The decreased expression of miR-187 was associated with increased tumor size (*t* = 4.106, *P* < 0.001) (Figure 1(b)) and TNM stage (*t* = 8.605, *P* < 0.001) (Figure 1(c)) in NSCLC patients. However, miR-187 downregulation was not correlated with other clinicopathological features, including patient sex, age, tumor organizational classification and tumor differentiation (*P* > 0.05) (Table 1), suggesting that miR-187 expression may be associated with NSCLC progress. NSCLC patients were divided into a high miR-187 expression group and a low expression group according to the relative expression of miR-187 in cancer tissues. The Kaplan–Meier analysis showed that lower expression of miR-187 predicted lower overall survival rate (log-rank *P* = 0.003) (Figure 1(d)), which indicated that a lack of miR-187 is predictive of a poor prognosis in NSCLC patients. The expression of miR-187 was also downregulated in all four NSCLC cancer cell lines (A549, H1975, NCI-H460 and SPC-A-1) compared to human normal lung epithelial cell 16HBE (Figure 1(e)). The downregulation was particularly notable in A549 (*P <*0.001) and SPC-A-1 (*P <*0.001) cells, which displayed results similar to those of the tissue samples. The above data suggested that miR-187 might be one potential critical inhibitory factor involved in NSCLC progression.

**Table 1.** Association of miR-187 expression with clinicopathological characteristics in NSCLC.

Clinicopathological characteristics	*N*	miR-187 expression	*P*-value
NSCLC	60	0.60 ± 0.27	0.000
Normal lung tissues	60	1.13 ± 0.30	
Sex			
Male	35	0.60 ± 0.26	0.763
Female	25	0.58 ± 0.29	
Age, years			
≤65	21	0.62 ± 0.25	0.610
>65	39	0.58 ± 0.29	
Histology			
Adenocarcinoma	31	0.56 ± 0.23	0.318
Squamous cell	29	0.63 ± 0.32	
Differentiation			
High–middle	41	0.61 ± 0.56	0.525
Low	19	0.29 ± 0.23	
Tumor size			
≤3 cm	35	0.70 ± 0.27	0.000*
>3 cm	25	0.45 ± 0.21	
AJCC stage			
I-II	37	0.74 ± 0.25	0.000*
III-IV	23	0.37 ± 0.11	

Note: *p < 0.05.

### Mir-187 overexpression inhibits proliferation and colony formation of NSCLC cells

**Figure 2.**
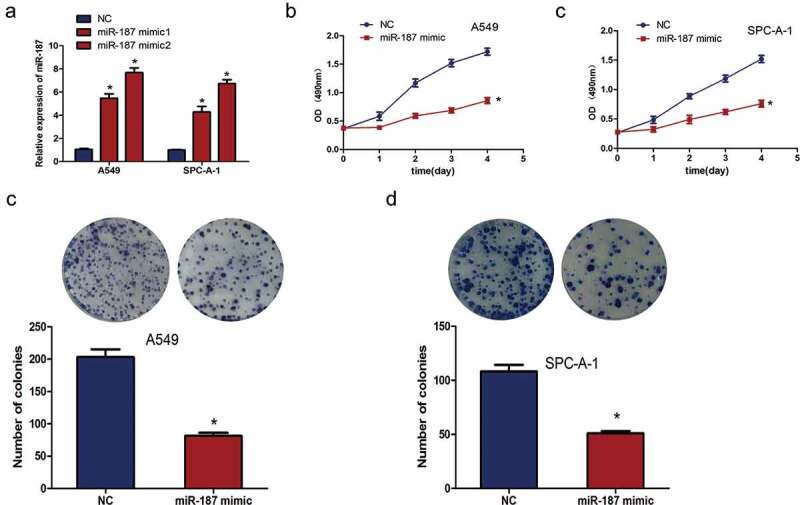
**miR-187 overexpression inhibits growth and colony formation ability of NSCLC cells**. (a) qRT-PCR analysis shows that miR-187 mimic transfection increases the expression of miR-187 in A549 and SPC-A-1 cells, and miR-187-mimic2 had a more obvious effect on increasing miR-187 expression. (b) MTS assay illustrates that miR-187 transfection significantly suppresses A549 cellular proliferation. (c) MTS assay illustrates that miR-187 transfection significantly suppresses SPC-A-1 cellular proliferation. (dC) Colony formation assay illustrates that miR-187 transfection significantly suppresses the cellular colony formation of A549 cells. (e) Colony formation assay illustrates that miR-187 transfection significantly suppresses cellular the colony formation of SPC-A-1 cells; *p < 0.05.

To further study the potential biological functions of miR-187 in NSCLC. The expression of miR-187 was increased in A549 and SPC-A-1 cells with two mimics, both of which induced high miR-187 expression levels; miR-187-mimic2 had a more obvious effect on increasing miR-187 expression than miR-187-mimic1 (*P* < 0.05) (Figure 2(a)). The reduced expression of miR-187 is related with increased tumor size and TNM stage, so an MTS assay was used to investigate the impact of miR-187 on cell proliferation. The results showed that miR-187 overexpression significantly decreased cell proliferation (*P* < 0.05) (Figure 2(b,c)). When transfected with miR-187 mimic in A549 and SPC-A-1 cells, the colonies of the A549 (Figure 2(d)) and SPC-A-1 (Figure 2(e)) cells were reduced significantly (*P* < 0.01). This result indicates that miR-187 has an anti-proliferation effect on cancer cells *in vito*.

### Mir-187 overexpression induces cell cycle arrest

**Figure 3.**
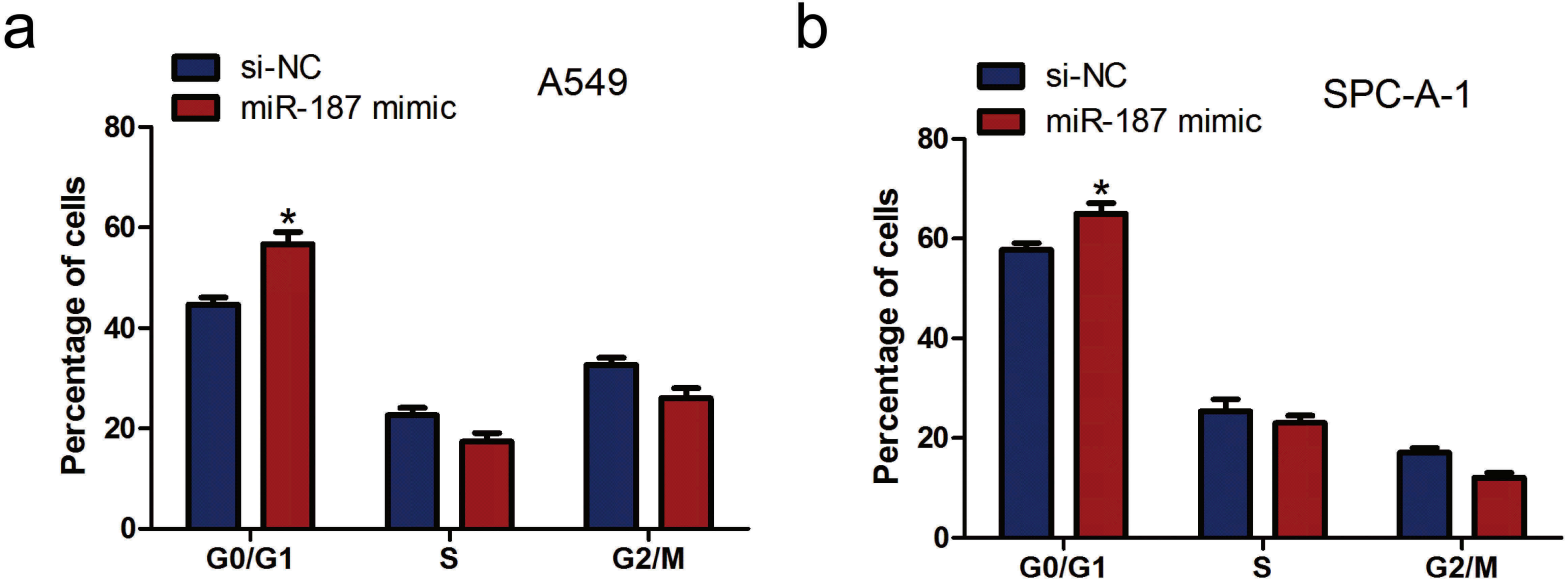
**miR-187 overexpression induces the cell cycle of NSCLC cell arrests**. (a) miR-187 transfection significantly increases the proportion of A549 cells in the G0/G1 phase, as assessed by FACS. (b) miR-187 transfection significantly increases the proportion of SPC-A-1 cells in the G0/G1 phase, as assessed by FACS; *p < 0.05.

To further investigate whether the inhibition of A549 and SPC-A-1 cell proliferation is caused by cell cycle arrest, the effect of miR-187 on cell cycle progression was analyzed using PI staining and flow cytometry. The results revealed that the number of A549 and SPC-A-1 cells in the G0/G1 phase was higher in the miR-187 mimic-transfected group than the NC group (*P* < 0.05) (Figure 3(a,b)). It demonstrated miR-187 induced cell cycle arrest at the G0/G1 phase.

### FGF9 is a target of mir-187 and is regulated by mir-187

**Figure 4.**
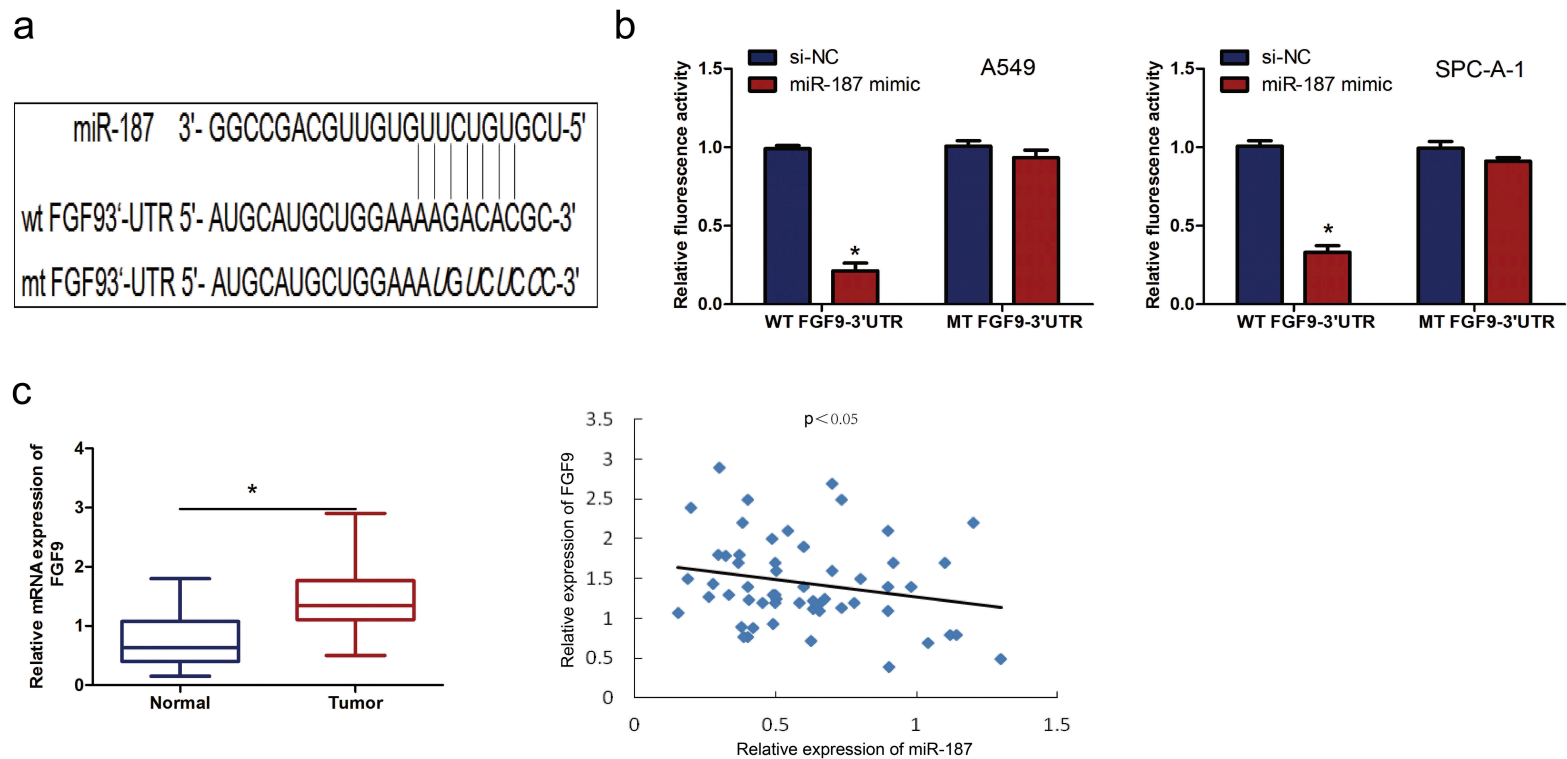
**FGF9 is a target of miR-187 and is regulated by miR-187**. (a) The binding sites of miR-187 within FGF9 3ʹ-UTR. (b) Luciferase reporter assay showed that miR-187 mimic transfection represses the luciferase activity of WT FGF9-3ʹUTR reporter in A549 and SPC-A-1 cells. (c) qRT-PCR analysis shows that miR-187 expression is higher in NSCLC tissues than paired normal tissues. (d) There is an inverse expression correlation between miR-187 and FGF9 mRNA in NSCLC tissues by qRT-PCR analysis. (e) miR-187 mimic transfection significantly inhibits the mRNA expression of FGF9 in A549 and SPC-A-1 cells; *p < 0.05.

MiRNAs regulate gene expression by targeting the mRNA 3ʹ-UTR; Liang *et al.* [21]reported that FGF9 had a complementary sequence for miR-187 binding (Figure 4(a)). Furthermore, the luciferase reporter assay showed that miR-187 mimic transfection repressed luciferase activity of FGF9-WT reporter in both A549 and SPC-A-1 cells (*P* < 0.01), indicating the direct interaction of miR-187 with FGF9 3ʹ-UTR (Figure 4(b)). Then, the expression levels of FGF9 in 60 NSCLC patient samples and paired normal tissues were examined by using qRT-PCR. The results showed that FGF9 expression was significantly upregulated in NSCLC tissues compared to normal tissues (*P* < 0.01) (Figure 4(c)). Furthermore, the expression of FGF9 was inversely correlated with miR-187 expression in NSCLC cancer tissues (*P* < 0.05, *r* = −0.424) (Figure 4(d)). Moreover, the miR-187 mimic significantly inhibited the mRNA expression of FGF9 in A549 and SPC-A-1 cells (*P* < 0.01) (Figure 4(e)). These data demonstrated that miR-187 plays an inhibitory role by targeting FGF9 in NSCLC.

### Mir-187 overexpression affected cell cycle protein expression

**Figure 5.**
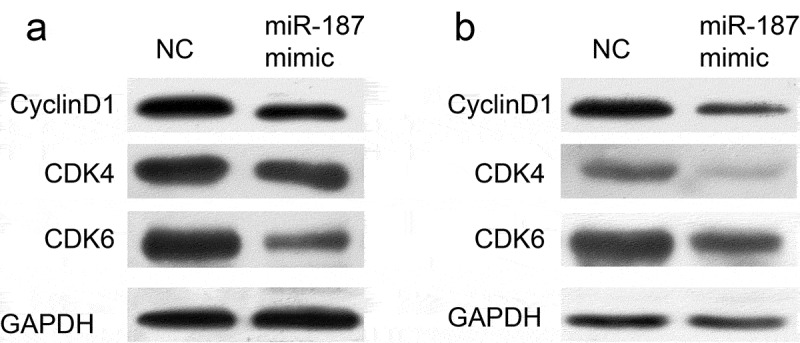
The effect of miR-187 on cell cycle protein expression. miR-187 overexpressed reduced the protein levels of cyclin D1, CDK4 and CDK6 in A549 and SPC-A-1 cells.

miR-187 overexpression induced cell cycle arrest at the G0/G1 phase, while the cell cycle transition is controlled by different cyclin proteins, and cell cycle-dependent kinase (CDKs), cyclin D1, CDK4 and CDK6 play a vital role in the G0/1-S transition [22]; thus, the effect of miR-187 on cyclin D1, CDK4 and CDK6 was examined. The overexpression of miR-187 in A549 and SPC-A-1 cells caused a marked decrease in the cell cycle-related protein level of cyclin D1, CDK4 and CDK6 (Figure 5).

### *Mir-187 overexpression inhibits growth of NSCLC cells* in vivo

**Figure 6.**
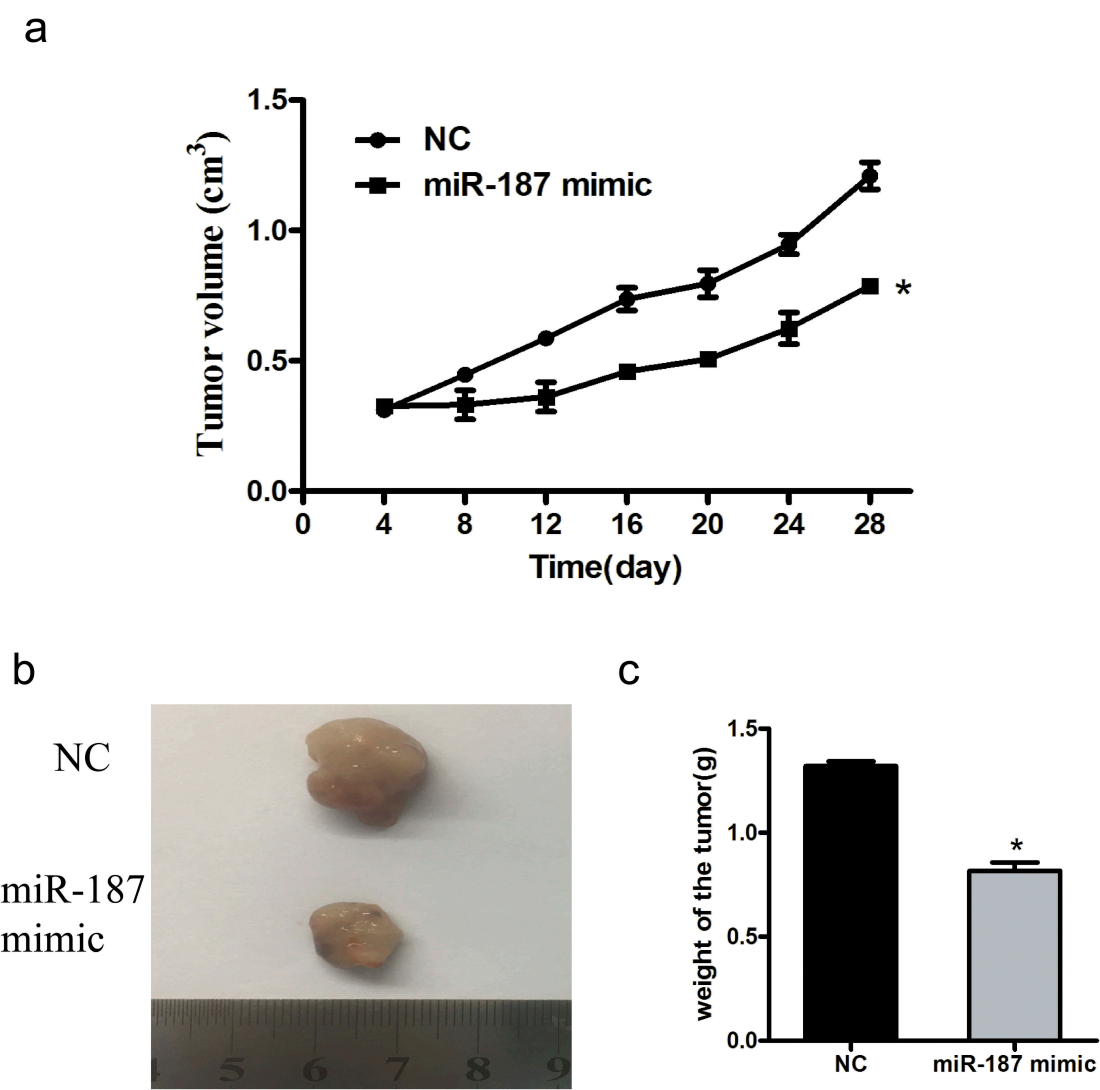
The effect of miR-187 on the growth of NSCLC cells *in vivo.* Nude mice xenografts illustrate that miR-187 transfection significantly suppresses the growth of A549 cells *in vivo*. (a) Growth curve of tumor xenografts. (b) Wet weights of tumor xenografts; *p < 0.05.

A nude mice xenograft assay was performed to confirm the *in vitro* data. The results demonstrated that the tumor xenografts following implantation of miR-187 mimic-infected cells grew significantly slower, compared with NC group (Figure 6(a)). And the mean weight of tumors from the nude mice in the miR-187 mimic group was lower than that of the mice in the NC group (Figure 6(b)).

## Discussion

NSCLC has the highest incidence and mortality rates among cancers in men and women; air pollution, smoking, eating habits, occupation, etc., are risk factors for NSCLC [23–25]. Early-stage NSCLC patients lack obvious symptoms, and more than half of the patients are diagnosed at an advanced stage with metastasis of distant organs [26]. Despite the rapid development of clinical medicine and experimental oncology, the overall five-year survival rate of NSCLC patients remains low [27]. Therefore, it is particularly important to find molecular targets for the treatment and outcome prediction of NSCLC. miRNAs are reported to serve as diagnostic or prognostic biomarkers in various cancers [28,29]. Furthermore, miRNAs can be detected accurately by various techniques, such as next-generation sequencing, microarray and qRT-PCR [30,31], among others. The discovery of miRNAs has provided novel insight into the molecular mechanisms and treatment of cancer. Increasing research has revealed that miRNAs including miR-758, miR-384, miR-148b, miR-520b and many other miRNAs [12,14,32] may affect oncogenes or tumor suppressors that are involved in NSCLC development and progression.

miR-187 is a recently identified cancer-associated miRNA. However, the expression level of miR-187 varies in different cancer tissues, and accumulating evidence showed that it may serve as tumor suppressors or oncogenes. Chen *et al.* [17] reported that miR-187 expression is significantly downregulated in gastric cancer and that the expression of miR-187 correlates with cell differentiation, TNM staging and poor prognosis in patients. However, miR-187 expression is significantly increased in plasma of OSCC patients, and miR-187 increased OSCC cell oncogenicity and xenograft metastasis in mice. These data indicate that miR-187 plays an important role in cancer development [18]. Cai *et al.* [33] found that miR-187 promotes cell migration and epithelial–mesenchymal transition (EMT) by targeting polymerase I and transcript release factor (PTRF) in NSCLC, and miR-187 potentially plays an oncogenic role in the progression of NSCLC.

In this study, the expression of miR-187 was measured in 60 paired NSCLC cancer and normal tissues by qRT-PCR, and the result showed that miR-187 expression was obviously lower in NSCLC cancer tissues than normal tissues. Consistent with the reports that the expression of miR-187-5p and miR-187-3p is decreased in NSCLC tissues [19,20], the expression of miR-187 in four NSCLC cells was significantly lower than that in 16HBE human normal lung epithelial cell. miR-187 expression downregulation was predictive of a poor prognosis associated with pathological grade, clinical stage, nodal metastasis and increased tumor size in gastric cancer patients, demonstrating that miR-187 plays a tumor-suppressive role [17]. Similarly, miR-187 expression levels are associated with increased tumor size and TNM stage of NSCLC. Furthermore, the reduced expression of miR-187 predicted a lower overall survival rate. Since miR-187 expression was downregulated in NSCLC tissues and cell lines, the molecular function of miR-187 in NSCLC to explore. A miR-187 mimic was transfected into cells to overexpress miR-187, and then, the proliferation of the cells was measured by an MTS assay. The results indicated that miR-187 overexpression inhibited NSCLC cell proliferation, which is consistent with the results of Xiao *et al.* [34] showing that miR-187 inhibits osteosarcoma cell proliferation *in vitro* and *vivo*, and Mao *et al.* [19] who reported that the overexpression of miR-187-5p inhibits the growth and metastasis of NSCLC cells. Colony formation assay indicated that miR-187 had an anti-clonogenic effect on cancer cells. In view of the above findings, miR-187 is speculated to function as a tumor suppressor in NSCLC, and the downregulation of miR-187 expression may promote the progression and development of NSCLC. However, the reason why miR-187 inhibits NSCLC cell proliferation remains to be investigated.

However, the reason why miR-187 inhibits cell proliferation of NSCLC remains to be investigated. Overall, aberrant cell cycle regulation is likely to be an important factor [35]. Indeed, subsequent cell cycle analyses in the present study revealed that A549 and SPC-A-1 cells that were transfected with miR-187 mimic had an evident cell cycle arrest at the G0/G1 phase. The main molecular mechanism of miRNA function is indirect regulation of target gene expression via the inhibition of protein synthesis or direct cleavage of the mRNA according to the degree of complementarity with the 3ʹuntranslated region of the target gene [8,9]. Lin *et al.* [36] demonstrated that miR-187 inhibits the cell proliferation, migration and invasion and promotes apoptosis by targeting human papillomavirus 16 E6 in cervical cancer cells. Furthermore, Cui *et al.* [37]reported that miR-187 regulated B7H3 (immune checkpoint molecule) expression by either direct or indirect pathways in many types of cancer and that this pathway may play an important role in the migration of tumor cells. miR-187 has a different function in NSCLC, possibly due to the regulation of different target genes. Liang *et al.* [21] revealed that overexpression of miR-187 could inhibit the proliferation and increase the apoptosis of human cervical cancer cells by targeting fibroblast growth factor 9 (FGF9). FGF9 is a well-known oncogenic gene in human cancers including ovarian cancer, hepatocellular carcinoma, NSCLC and other cancers [38–40]. FGF9 increases cell proliferation by activating cell cycle pathway proteins, including cyclin D1, cyclin E1 and cyclin A1 in mouse Leydig tumor cells [41]. Whether FGF9 target genes are responsible for the biological effects in NSCLC is currently unknown. The data demonstrated that FGF9 is the target gene of miR-187 in A549 and SPC-A-1 cells. Moreover, miR-187 mimic transfection significantly inhibited the mRNA expression of FGF9 and the protein expression of cyclin D1, CDK4 and CDK6. It is likely that the cell cycle arrest, which resulted in the cell proliferation reduction induced by miR-187, is influenced by targeting FGF9 and the consequent effect on cyclin D1, CDK4 and CDK6.

In conclusion, the present study results demonstrate that miR-187 expression is significantly decreased in NSCLC tissues and cell lines. Moreover, the decreased expression of miR-187 is associated with the increased tumor size and TNM stage and poor overall survival rate in NSCLC patients. The data of the present study also indicate that miR-187 could inhibit cell proliferation and could induce cell cycle arrest at the G0/G1 phase by targeting FGF9. The study provides evidence for the potential effectiveness of miR-187 as a biomarker and therapeutic target for NSCLC.

## Highlights

1. miR-187 was downregulated in NSCLC tissues and correlated with prognosis.
2. miR-187 inhibited NSCLC cell proliferation and induced cell cycle arrest.
3. miR-187 inhibited cell cycle protein cyclinD1, CDK4 and CDK6.
